# Toll‐like receptor 2 activation induces C–C chemokine receptor 2‐dependent natural killer cell recruitment to the peritoneum

**DOI:** 10.1111/imcb.12379

**Published:** 2020-09-09

**Authors:** Ian D Haidl, Dihia Meghnem, Thomas B Issekutz, Jean S Marshall

**Affiliations:** ^1^ Dalhousie Human Immunology and Inflammation Group Dalhousie University Halifax Nova Scotia Canada; ^2^ Department of Microbiology and Immunology Dalhousie University Halifax Nova Scotia Canada; ^3^ Department of Pediatrics Dalhousie University Halifax Nova Scotia Canada; ^4^ Department of Pediatrics Dalhousie University Halifax Nova Scotia Canada; ^5^ IWK Health Centre Halifax Nova Scotia Canada

**Keywords:** Inflammation, innate immunity, pattern recognition receptor, lipopeptide

## Abstract

Natural killer (NK) cells are innate effector cells with critical roles not only in tumor immunosurveillance and viral immunity, but also in bacterial and fungal infections. Toll‐like receptor 2 (TLR2) can be important in the early and sustained immune responses to pathogens and tumors through the induction of cytokines and chemokines that recruit and activate immune effector cells. We investigated the role of TLR2 activation in NK cell recruitment with a view to informing approaches to induce or regulate peritoneal NK cell responses therapeutically. Peritoneal injection of TLR2 activators, including peptidoglycan and the lipopeptides FSL‐1 and Pam_3_CSK_4_, resulted in NK cell recruitment after 16 h with increased NK cell numbers maintained for 48 h. TLR2 activators induced large amounts of CCR2 ligands, but much smaller amounts of CCR5 and CXCR3 ligands. Consistent with this observation, NK cell migration was abrogated in CCR2‐deficient mice after peritoneal FSL‐1 injection. Adoptive transfer of CCR2‐deficient NK cells prior to peritoneal FSL‐1 activation confirmed a cell‐intrinsic component of CCR2‐mediated NK cell migration. TLR2 activation did not induce an activated NK cell phenotype, but significant changes included an increase in the KLRG1^+^ subset and decreased NKG2D expression. Although not activated *in vivo*, peritoneal NK cells could be activated by interleukin (IL)‐12 and IL‐18 *ex vivo* to express CD69 and interferonγ. These data demonstrate that TLR2‐mediated immune activation is a potent inducer of NK cell recruitment via a CCR2‐dependent mechanism and that NK cells recruited by this mechanism can respond to additional signals to exert effector cell functions.

## Graphical abstract

In this study, we showed that induction of inflammation in the peritoneal cavity by Toll‐like receptor 2 (TLR2) agonists induces chemokine production with very high levels of the CCR2 ligands CCL2 and CCL7. Natural killer (NK) cells are recruited to the peritoneum after TLR2‐induced inflammation in a CCR2‐dependent manner, but do not acquire an activated phenotype. Recruited NK cells can be activated by interleukin (IL)‐12 and IL‐18 to express CD69 and interferonγ.

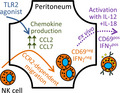

## Introduction

Toll‐like receptor 2 (TLR2) is a pattern recognition receptor that is critical in mediating immune responses ranging from tolerance induction to controlling infection by multiple pathogens and mediating tumor regression.^1^, ^2^, ^3^ TLR2 is expressed on several cell types and pairs with either TLR1 or TLR6 to form a cell surface receptor capable of recognizing several bacterial molecules, fungal cell wall components and certain viral proteins. TLR2 also mediates responses to endogenous alarmins such as high‐mobility group protein B1.^4^, ^5^, ^6^ TLR2 activation classically initiates signaling through the MyD88 adaptor protein which results in nuclear factor‐κB activation with subsequent chemokine and cytokine production.^4^, ^6^ The physiological consequences of TLR2 activation vary according to the precise receptor(s) and cell type involved, but the result is often cell activation to aid in direct pathogen or tumor cell killing and the recruitment of additional immune effector cells. The importance of TLR2 is seen in TLR2‐deficient mice, which are highly susceptible to infection by bacteria including *Staphylococcus aureus* and *Streptococcus pneumoniae*,^7^, ^8^ parasites such as *Toxoplasma gondii*,^9^ fungi such as *Candida albicans* ^10^ and have decreased responses to certain viral infections including respiratory syncytial virus.^11^

TLR2 activators have been investigated as anti‐infective and antitumor therapeutics owing to their immunostimulatory properties.^3^, ^12^ In previous studies, we noted that natural killer (NK) cells, along with T cells, were enriched following therapeutic delivery of TLR2 activators to tumor sites in mice.^13^ TLR2 activators have also previously been shown to directly activate NK cells.^14^ NK cells have been extensively studied in immune responses to tumors and pathogenic infections, where they utilize direct cytotoxicity, antibody‐dependent cellular cytotoxicity and/or the production of cytokines such as interferon (IFN)γ to clear pathogens or reduce tumor load.^15^, ^16^ NK cells also possess properties previously attributed only to adaptive immune cells, such as antigen specificity, clonal expansion and the development of long‐lived memory cells.^17^ NK cells have been demonstrated to play a role in more complex immunoregulatory processes such as the amplification or inhibition of T‐cell responses. This regulation can be mediated by multiple mechanisms including NK cell killing of T cells or dendritic cells or via NK cell cytokine production to alter T‐cell or dendritic cell function or via effects on other cell types. The resulting changes can affect T‐cell immunity, alter disease pathology and influence T‐ and B‐cell memory generation.^18^, ^19^ These regulatory NK cell functions are known to modulate disease in experimental models and human patients involving conditions such as cancer, autoimmune diseases, atherosclerosis and tissue fibrosis.^20^, ^21^

Given the multiple roles of NK cells as direct effector cells and regulators of the innate and adaptive immune responses, initial NK cell recruitment may be a key event in determining the immune response at sites of infection, inflammation and tumor growth. Because these sites also commonly have increased levels of exogenous or endogenous TLR2 ligands, we initiated an analysis of NK cell recruitment in response to TLR2 activation. Depending on the cause and site of inflammation, it is known that NK cell recruitment can be mediated by several chemokine receptors, including CCR2, CCR5 and CXCR3.^22^, ^23^, ^24^ The mouse peritoneal cavity is commonly utilized as a relevant model system for examining responses to bacterial and viral infections and tumors. In addition, humans can develop infectious peritonitis in response to intestinal perforation and the peritoneum is a common site of secondary tumor development in colon and ovarian cancer.^25^, ^26^ We therefore sought to determine whether mouse peritoneal NK cell recruitment could be induced by TLR2 activation and investigate which chemokine receptors mediate this process.

## RESULTS

### NK cell recruitment to the peritoneum is induced by TLR2 activation

**Figure 1:**
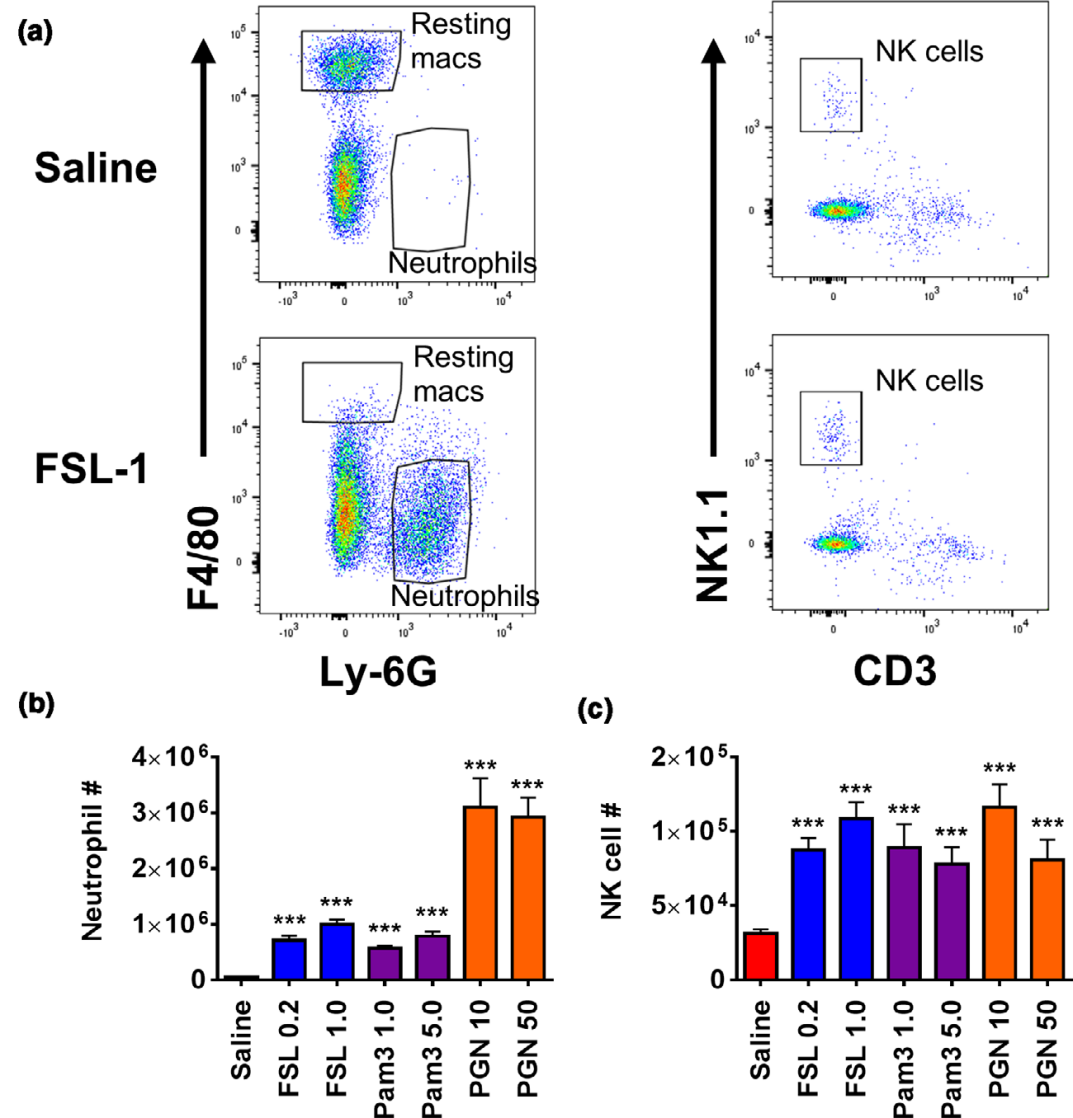
Natural killer (NK) cell and neutrophil recruitment in response to FSL‐1, Pam_3_CSK_4_ and peptidoglycan (PGN). C57BL/6 mice were injected intraperitoneally with saline, FSL‐1 (0.2 or 1 μg), Pam_3_CSK_4_ (1 or 5 µg) or PGN (10 or 50 μg). After 16 h the peritoneal contents were harvested by lavage, the total cells were counted and the percentages of NK cells (lymphocyte gate; NK1.1^+^/CD3^–^) and neutrophils (CD11b^+^ gate; F4/80^–^, Ly6G^+^; as shown in **(a)** in each sample were identified by flow cytometric analysis. The graphs for **(b)** neutrophils and **(c)** NK cells show the means ± s.e.m., *n* = 9–40, with three to nine separate experiments for the different activation conditions. ***P < 0.001 *versus* saline‐treated control. Macs, macrophages.

To investigate the early peritoneal NK cell recruitment in response to activation by bacteria‐associated TLR2 activators, intraperitoneal injections were performed with FSL‐1 (a diacyl lipopeptide that activates TLR2/6), Pam_3_CSK_4_ (a triacyl lipopeptide that activates TLR2/1) and peptidoglycan (PGN; which activates TLR2, NOD2 and complement receptors). Analysis was performed 16 h after injection to identify peritoneal cell populations including neutrophils and NK cells (Figure 1a and Supplementary figure 1). This showed that all activators induced peritoneal inflammation with a significant increase in the number of neutrophils (Figure 1b). Interestingly, there was also a significant increase in peritoneal NK cell numbers (Figure 1c), with the NK cell number increased by 3.47 ± 0.35‐, 2.49 ± 0.36‐ and 3.72 ± 0.49‐fold after treatment with 1 µg FSL‐1, 5 µg Pam_3_CSK_4_ and 10 µg PGN.

**Figure 2:**
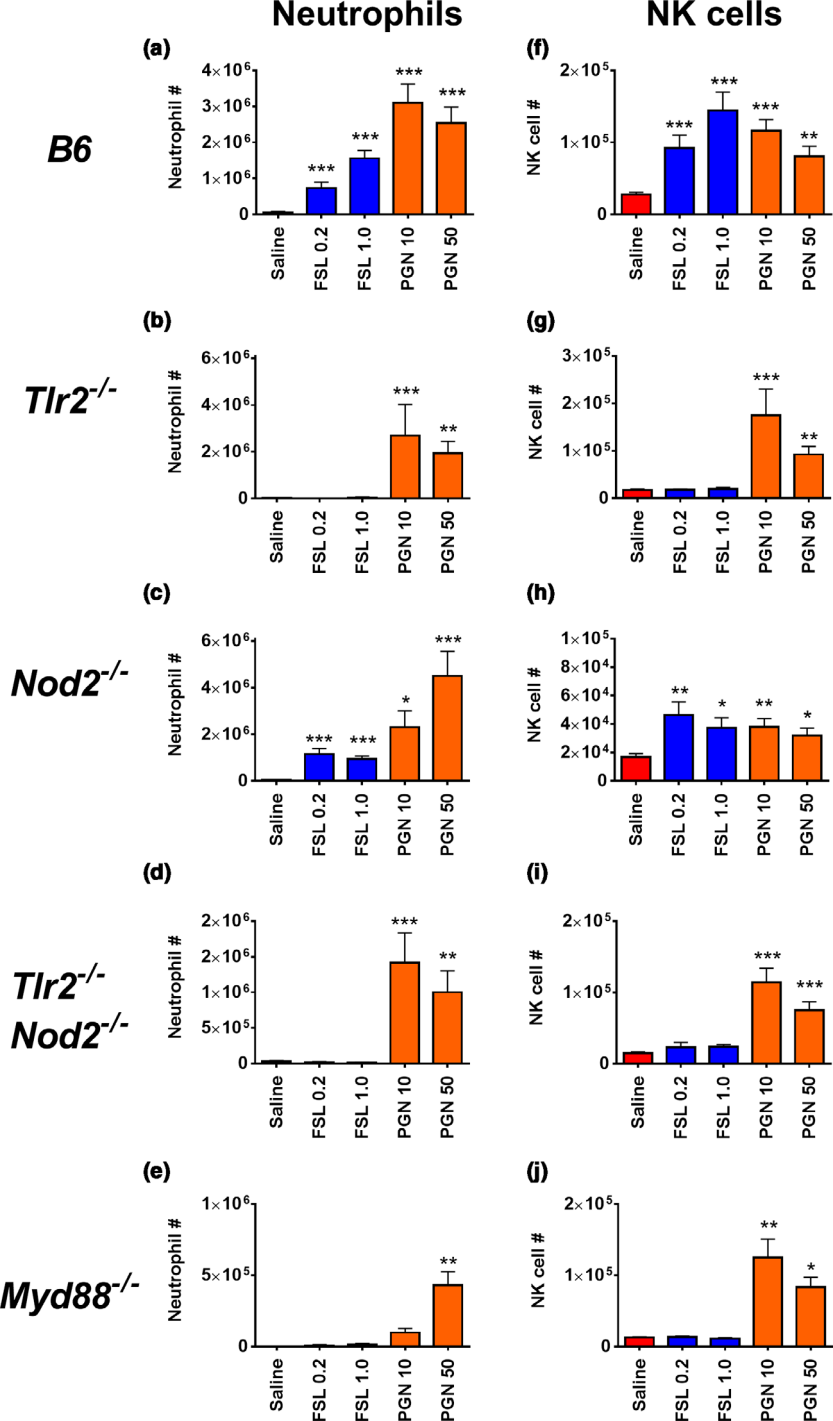
Natural killer (NK) cell and neutrophil recruitment in response to FSL‐1 and peptidoglycan (PGN) in the absence of TLR2, NOD2, both TLR2 and NOD2 and MyD88. Mice were injected intraperitoneally with saline, FSL‐1 (0.2 or 1 μg) or PGN (10 or 50 μg). After 16 h the peritoneal contents were harvested by lavage, the total cells were counted and the percentages of NK cells and neutrophils in each sample were identified by flow cytometric analysis to calculate the number of **(a–e)** neutrophils and **(f–j)** NK cells. The graphs show the means ± s.e.m., *n* = 4– 20, with two to five separate experiments for the different activation conditions. *P < 0.05; **P < 0.01, ***P < 0.001 *versus* saline‐treated control of each strain. TLR2, Toll‐like receptor 2.

To assess the degree to which the observed NK cell recruitment was dependent on TLR2 signaling, we analyzed the peritoneal recruitment of NK cells in response to FSL‐1 and PGN in mice deficient for TLR2, NOD2, both TLR2 and NOD2 or MyD88. As expected from previous reports,^8^ lipopeptide required the presence of both TLR2 and MyD88 to induce inflammation as indicated by the lack of neutrophil recruitment in the absence of TLR2 or MyD88 (Figure 2a–e). NK cell recruitment was also curtailed in TLR2‐ or MyD88‐deficient animals, indicating that TLR2‐specific NK cell recruitment likely follows classical TLR2 activation and signaling pathways (Figure 2f–j). PGN activates multiple receptor systems and retained the ability to induce inflammation in the absence of TLR2 or NOD2 alone. Even in double knockouts (*Tlr2^–/–^/Nod2^–/–^*) and MyD88‐deficient mice, significant, though reduced, PGN‐mediated inflammation was evident.

### Peritoneal chemokine production induced by TLR2 activation

**Figure 3:**
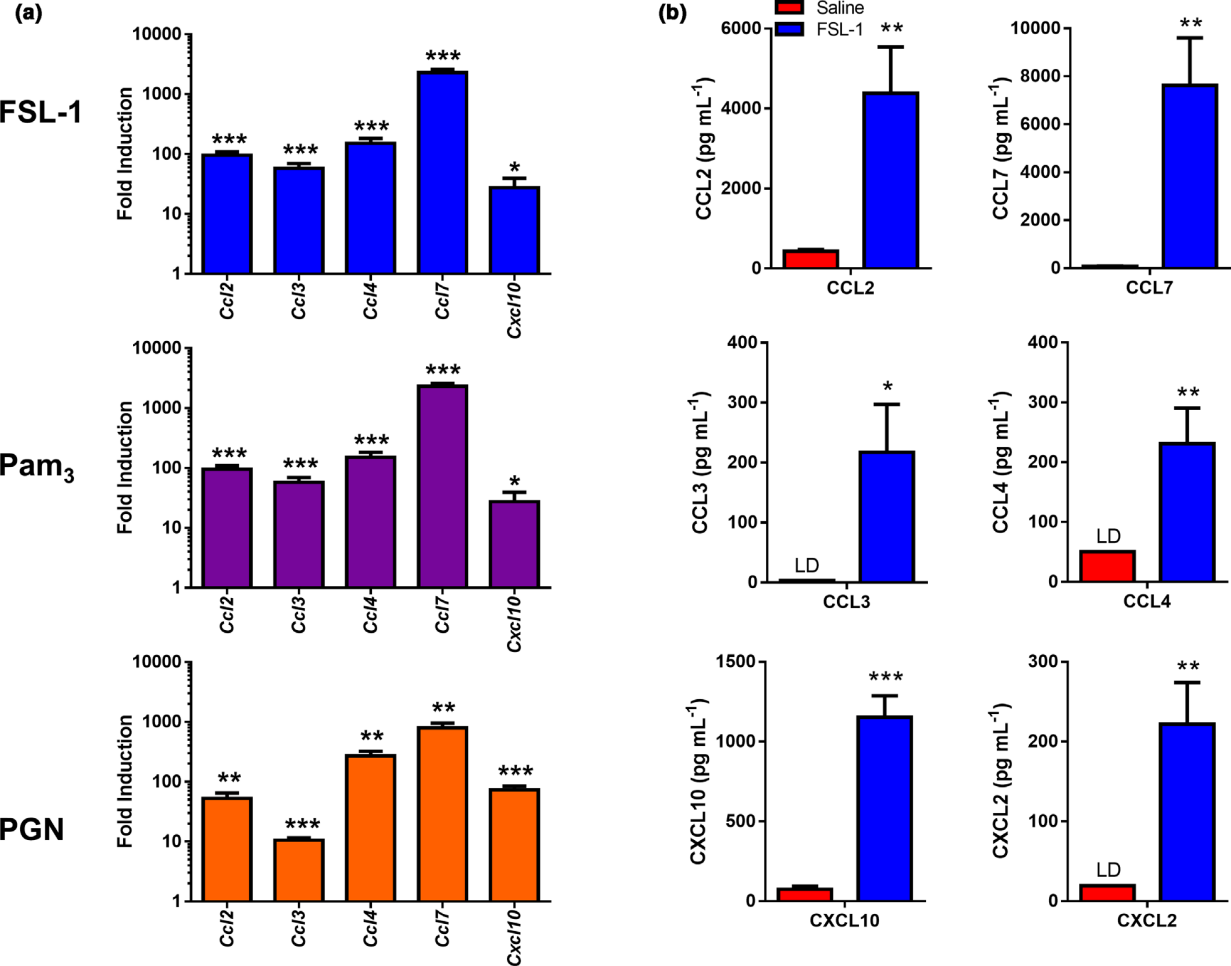
Chemokine gene expression in the peritoneum following treatment with FSL‐1, Pam_3_CSK_4_ or peptidoglycan (PGN). The peritoneal contents of C57BL/6 mice were harvested 16 h following intraperitoneal (i.p.) injection **(a)** with saline, 1 μg FSL‐1, 5 µg Pam_3_CSK_4_ (Pam_3_) or 10 µg PGN. RNA from the peritoneal cells was prepared and the gene expression of several chemokines was analyzed by quantitative PCR. The fold induction of each gene by FSL‐1 treatments *versus* saline is shown, but statistics were performed using the normalized expression values for comparisons to the respective saline values. *n* = 5 or 6 in two separate experiments. *P < 0.05; **P < 0.01; ***P < 0.001. **(b)** The cell‐free lavage fluid harvested 16 h following i.p. injection of 1 µg of FSL‐1 was analyzed by Luminex multiplex analysis for multiple chemokines. The graphs show means ± s.e.m., *n* = 5–10 in two or three separate experiments. Statistical significance was determined by comparisons of FSL‐1 with the respective saline value. *P < 0.05; **P < 0.01; ***P < 0.001; LD, limit of detection.

NK cell recruitment into the inflamed peritoneum, and subsequent NK cell activation, are likely to be controlled by the production of selected chemokines and cytokines. To analyze the expression of these mediators in TLR2‐specific peritoneal inflammation, we utilized peritoneal cavity cells from FSL‐1‐treated mice to perform a PCR array analysis of gene expression. The genes that were most significantly upregulated in response to FSL‐1 included those encoding several chemokines implicated in NK cell recruitment such as CCL2, CCL3, CCL4, CCL7 and CXCL10 (Table 1). Analysis of chemokine gene expression in response to Pam_3_CSK_4_ and PGN confirmed their significant upregulation in response to all three activators (Figure 3a). Interestingly, the expression of mRNAs for several mediators known to be involved in NK cell activation [e.g. interleukin (IL)‐2, IL‐12, IL‐15, IL‐18 and IFNα2] was unchanged or slightly downregulated following FSL‐1 injection (Table 1). In comparing the pattern of gene induction for the three TLR activators, the TLR2‐specific lipopeptides, Pam_3_CSK_4_ and FSL‐1, showed very similar levels of chemokine gene induction, while PGN, likely because of its potential to activate multiple pathways, was similar but showed some differences such as lower *Ccl3* induction. Therefore, for selective analysis of the TLR2‐induced aspects of peritoneal inflammation, further analysis focused on the response to FSL‐1. Analysis of peritoneal lavages from FSL‐1‐injected mice revealed significant production of chemokines associated with NK cell recruitment in addition to other chemokines such as the neutrophil chemoattractant CXCL2 (Figure 3b). In particular, CCL2 and CCL7, both CCR2 ligands, were produced at very high levels.

**Table 1:** Changes in peritoneal cavity cell gene expression after FSL‐1 treatment^a^

Gene	Fold upregulation
*Ccl7*	396.73
*Ccl12*	195.63
*Cxcl3*	164.51
*Ccl4*	69.17
*Il1rn*	61.48
*Il1b*	49.94
*Ccl3*	45.95
*Cxcl10*	42.87
*Cxcl1*	40.84
*Ccl2*	37.07
*Il12b*	1.27
*Il18*	1.02
*Il12a*	0.60
*Ifna2*	0.46
*Il2*	0.46
*Il15*	0.40

^a^ C57BL/6 mice were injected intraperitoneally with either saline or 1 μg FSL‐1. The peritoneal cavity cells were harvested 16 h later, RNA was prepared and complementary DNA (cDNA) produced. The cDNA was added to quantitative PCR (qPCR) array plates and qPCR was performed according to the manufacturer’s instructions. Data were normalized using five reference genes and the fold upregulation for each gene was calculated. The top 10 upregulated genes are shown in the upper rows of the table. The genes in the lower rows encode proteins that can be involved in natural killer cell activation that were not upregulated after FSL‐1 treatment.

### TLR2‐mediated peritoneal NK cell recruitment is dependent on CCR2

**Figure 4:**
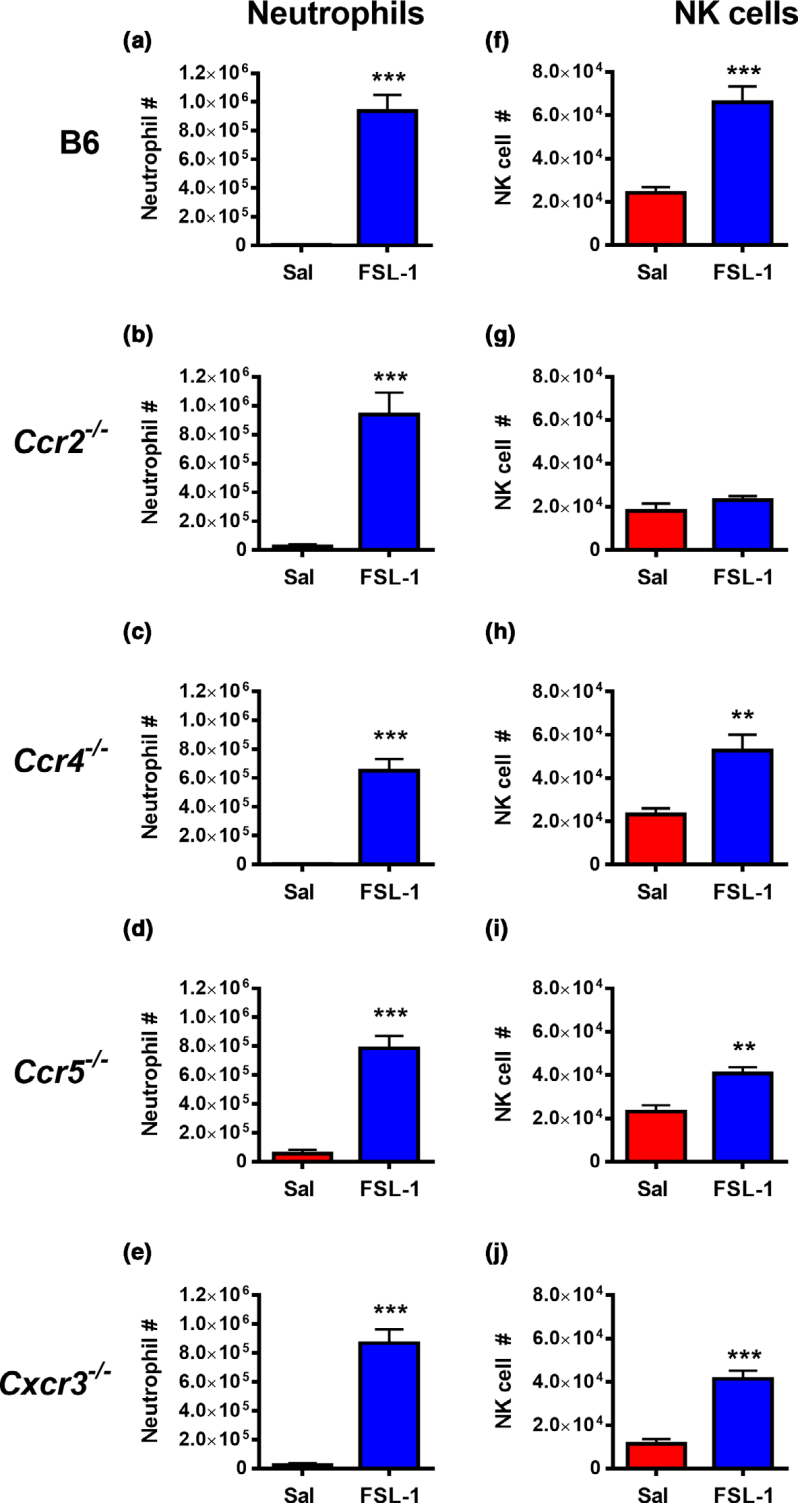
Natural killer (NK) cell and neutrophil recruitment in response to FSL‐1 in the chemokine receptor knockout strains *Ccr2^−/−^*, *Ccr4^−/−^_,_ Ccr5^−/−^* and *Cxcr3* *^−/−^*. Wild‐type mice or the chemokine receptor knockout strains indicated were injected intraperitoneally with saline (Sal) or FSL‐1 (1 μg). After 16 h, the peritoneal contents were harvested by lavage, the total cells were counted and the percentages of NK cells and neutrophils were identified by flow cytometric analysis to calculate the number of **(a–e)** neutrophils and **(f–j)** NK cells. The graphs show the means ± s.e.m., *n* = 7–15 in three to six separate experiments. **P < 0.01; ***P < 0.001 *versus* saline‐treated control in each strain.

Given that potential NK chemotactic ligands for CCR2, CCR5 and CXCR3 were upregulated in response to TLR2 activation, we evaluated the extent of FSL‐1‐induced NK cell recruitment in mice deficient for these receptors and in CCR4‐deficient mice. Although recruited NK cell numbers in CCR5‐ and CXCR3‐deficient mice were lower than in wild‐type animals, mice lacking CCR4, CCR5 and CXCR3 all exhibited significant migration of NK cells into the peritoneum in response to FSL‐1 (Figure 4f–j). By contrast, animals lacking CCR2 displayed a profound inhibition of NK cell recruitment (Figure 4g). Neutrophil responses in the absence of CCR2 were normal, indicating that FSL‐1 had induced an inflammatory response (Figure 4a–e).

**Figure 5:**
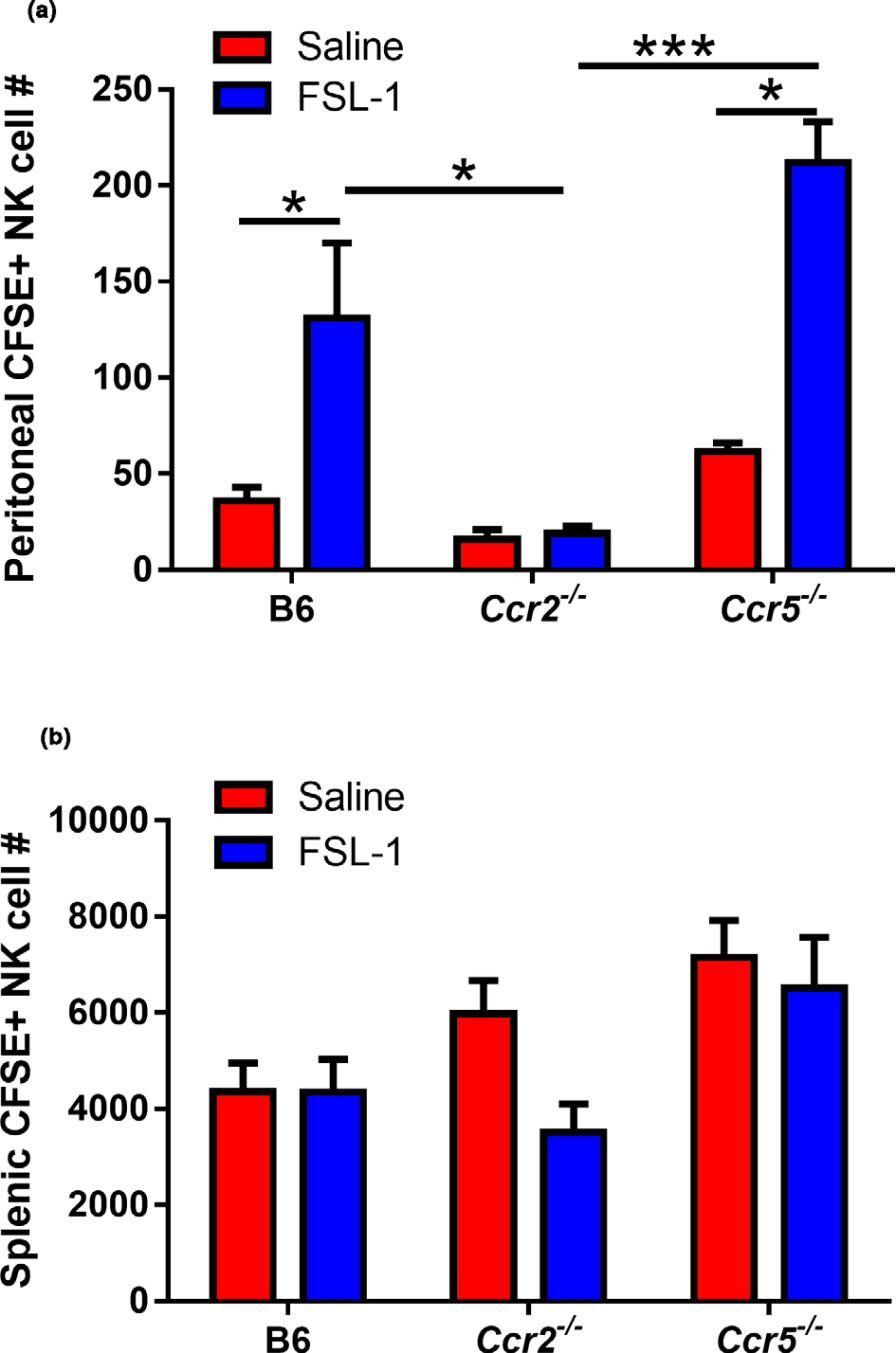
Natural killer (NK) cell recruitment in response to FSL‐1 following adoptive transfer of carboxyfluorescein succinimidyl ester (CFSE)‐labeled cells from C57BL/6, *Ccr2^−/−^* or *Ccr5^−/−^* mice. NK cells were enriched from the spleens of C57BL/6, *Ccr2^−/−^* or *Ccr5^−/−^* mice, labeled with CFSE and injected intravenously into C57BL/6 recipients. These mice were immediately injected intraperitoneally with saline or FSL‐1 (1 μg). After 16 h the peritoneal contents **(a)** and spleens **(b)** were harvested, the total cells were counted and cells were analyzed by flow cytometry to identify CFSE‐positive NK cells. The numbers shown have been normalized by calculating the number of CFSE^+^ NK cells per 10^5^ NK cells injected in each mouse. The graphs show the means ± s.e.m., *n* = 3– 8 derived from six enrichment experiments for C57BL/6 and two each for *Ccr2^−/−^* and *Ccr5^−/−^*. *P < 0.05; ***P < 0.001.

The decrease in FSL‐1‐induced peritoneal NK cell migration in CCR2‐deficient mice could have been a result of a lack of responsiveness intrinsic to the NK cells or potentially as a result of an altered peritoneal microenvironment caused by the lack of inflammatory monocyte recruitment.^29^ Therefore, we characterized the chemokine production in *Ccr2^–/–^* mice after FSL‐1 treatment. As previously reported,^30^ CCR2 ligands are present in higher amounts in the absence of their receptor CCR2 (Supplementary figure 2). Ligands for other potential NK cell chemotactic receptors CCR5 and CXCR3 were produced in significant quantities in Ccr2^–/–^ mice after FSL‐1 treatment, which indicated that these receptors were not mediating NK cell recruitment. To test for an NK cell‐intrinsic influence on recruitment, NK cells were enriched from C57BL/6, CCR2‐deficient or CCR5‐deficient spleens, carboxyfluorescein succinimidyl ester (CFSE) labeled and injected intravenously into C57BL/6 recipients prior to peritoneal FSL‐1 activation. After 16 h the spleens and peritoneal cells were harvested and analyzed for the presence of CFSE‐labeled NK cells (Supplementary figure 3). As shown in Figure 5, in the absence of inflammation there is very little migration to the peritoneum as expected.^31^ Following FSL‐1‐induced inflammation, the transferred CCR2‐deficient NK cells showed minimal migration to the recipient peritoneum. By contrast, significant numbers of NK cells from wild‐type or CCR5‐deficient animals migrated to the peritoneum after FSL‐1 activation (Figure 5a).

### Peritoneal NK cell phenotype following TLR2 activation

**Figure 6:**
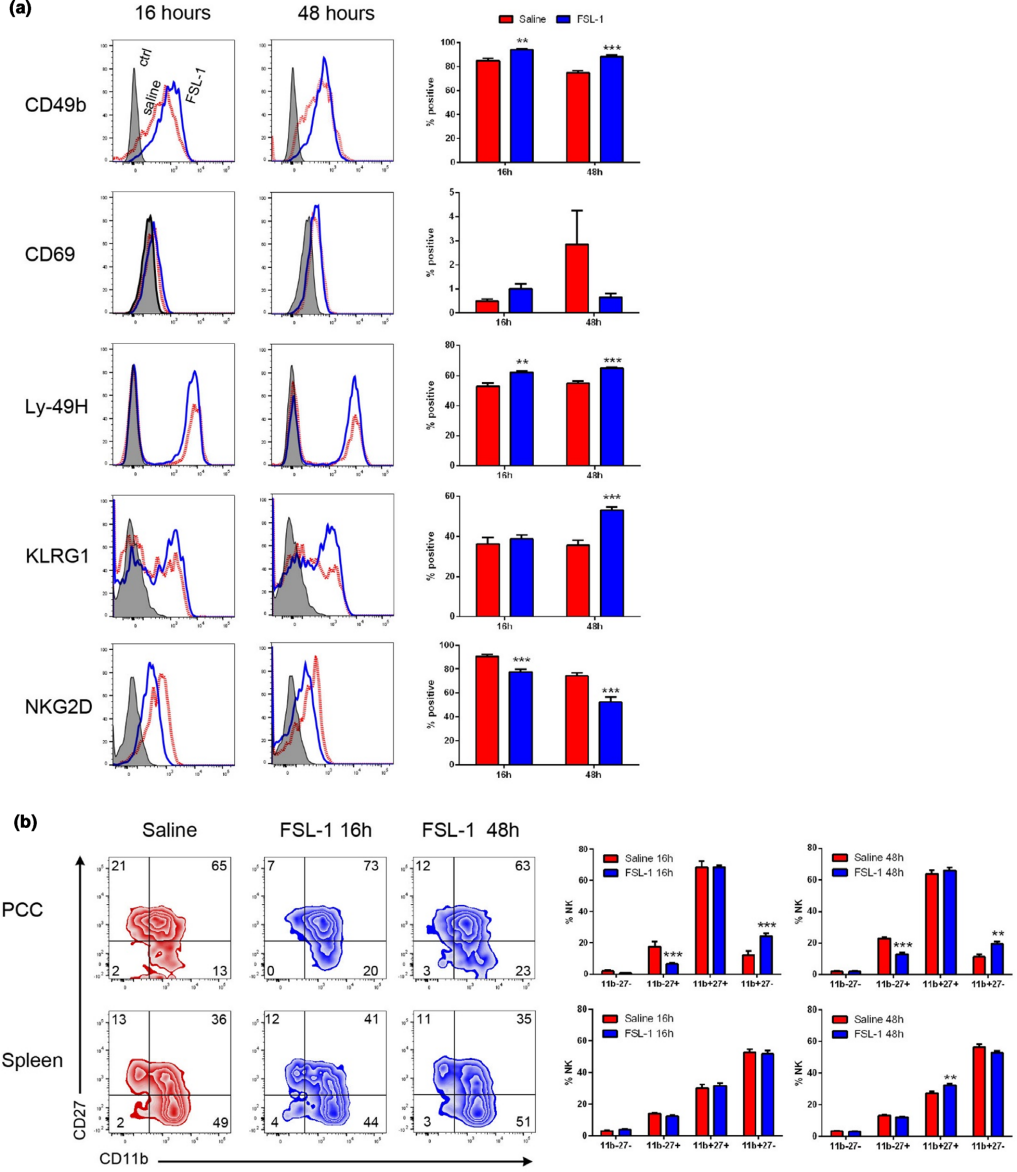
Natural killer (NK) cell phenotype 16 and 48 h after FSL‐1 activation. Mice were injected intraperitoneally with saline or FSL‐1 (1 μg). After 16 or 48 h peritoneal contents were harvested by lavage, the total cells were counted and the NK cells in each sample were identified by flow cytometric analysis. **(a)** The NK population was further analyzed for the expression of NK cell activation or subset markers. **(b)** The expression of maturation markers CD11b and CD27 is shown for peritoneal and splenic NK cells. The graphs show the means ± s.e.m. (16 h *n* = 8; 48 h *n* = 12) in two or three separate experiments. **P < 0.01, ***P < 0.001 *versus* saline‐treated control. Ctrl, control.

NK cell populations can vary significantly in the composition of phenotypically distinct NK cell subsets that reflect cells at different developmental stages, with variable functional capabilities, and at different states of activation. Therefore, we characterized the phenotype of the peritoneal NK cell population present following TLR2 activation. We first analyzed NK cell recruitment at up to 48 h after TLR2 activation by FSL‐1 and confirmed that the increase in NK cell numbers was sustained similar to 16 h postactivation (2.89 ± 0.31‐fold more than saline, *n* = 12). At 16 and 48 h after activation, the peritoneal NK cell population did not have a classical activated phenotype, showing no increase in CD69^+^ populations (Figure 6a), which is consistent with the lack of gene induction for several cytokines associated with NK cell activation (Table 1). Among the notable changes in the NK cell population was an increase in the KLRG1^+^ population at 48 h, which was unchanged at 16 h. NKG2D expression at 16 and 48 h was downregulated, possibly in response to NKG2D ligand expression following TLR activation.^32^

To assess the developmental stages of peritoneal NK cells, expression of CD11b and CD27 was determined to identify the four populations that proceed developmentally from the most immature CD11b^lo^CD27^lo^ to CD11b^lo^CD27^hi^ to CD11b^hi^CD27^hi^ and finally to CD11b^hi^CD27^lo^. The CD11b^hi^ populations have effector functions such as cytotoxicity and IFNγ secretion.^33^ Peritoneal NK cells were close to 70% CD11b^hi^CD27^hi^ cells and 10–15% were the more mature CD11b^hi^CD27^lo^ population (Figure 6b). This contrasts with the spleen, which has a greater proportion of CD11b^+^CD27^–^ than CD11b^+^CD27^+^ NK cells. TLR2 activation in the peritoneum decreased the minor population of immature CD11b^–^CD27^+^ NK cells at both 16 and 48 h, with a corresponding increase in the percentage of the mature CD11b^+^CD27^–^ NK cell population (Figure 6b). Taken together, the phenotypic analysis of peritoneal NK cells after TLR2‐induced inflammation suggests that although the NK cells are not fully activated, the resulting NK cell population can be responsive to ligands (based on NKG2D downregulation^32^) and shows moderate changes in subsets and maturation stages.

### Peritoneal NK cells can be activated *ex vivo* by IL‐12 and IL‐18

**Figure 7:**
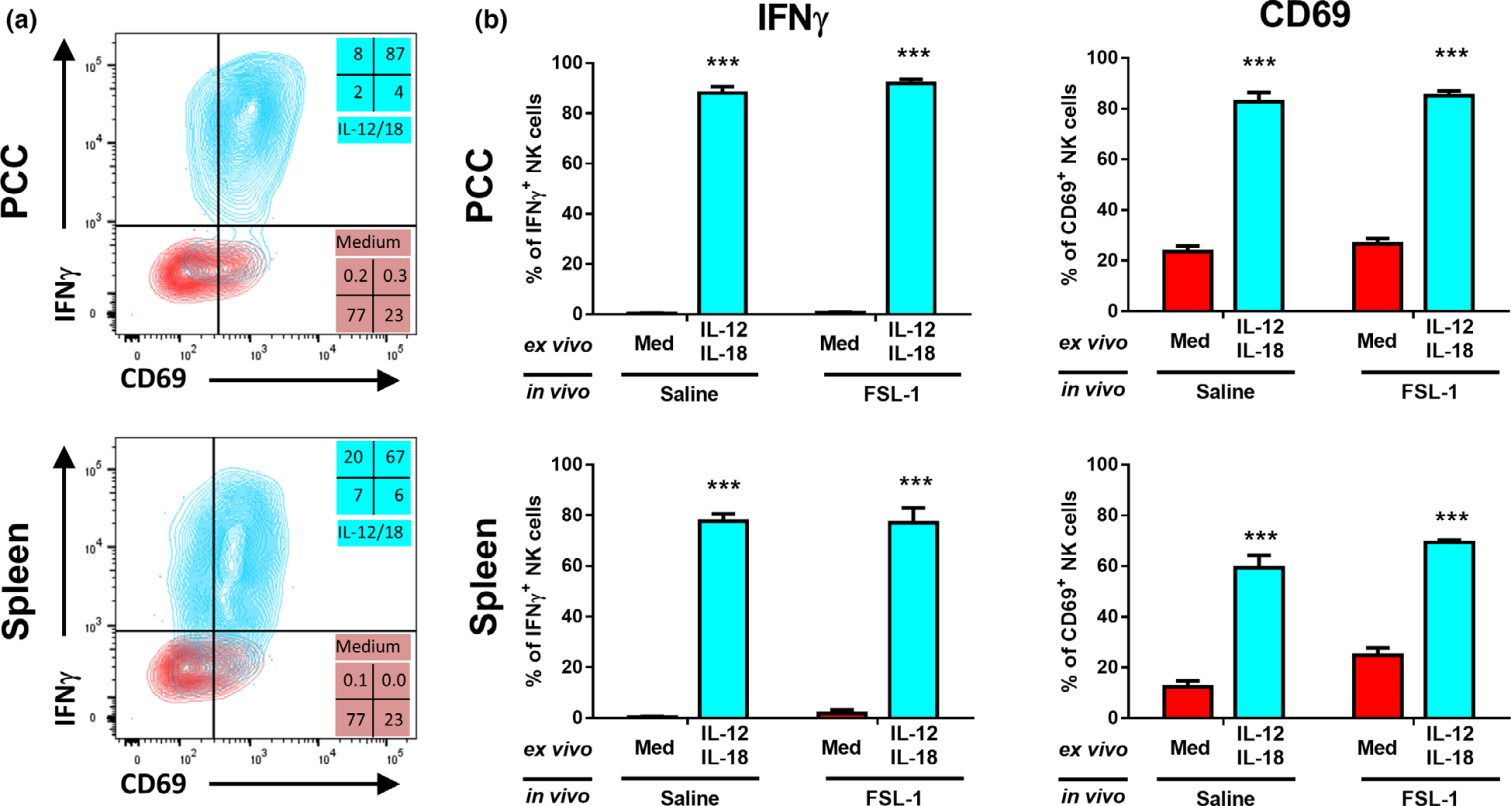
Peritoneal natural killer (NK) cells from control or TLR2‐activated mice can be activated by interleukin (IL)‐12 and IL‐18. Mice were injected intraperitoneally with saline or FSL‐1 (1 μg). After 16 h the peritoneal cells (PCC) and spleens were harvested and cultured for 1 h. Nonadherent cells were further cultured in plain medium (Med) or in the presence of IL‐12 and IL‐18 for 5 h before the addition of brefeldin A and monensin for the final hour of culture. The cells were harvested and stained with antibodies to NK1.1, CD3 and CD69 before intracellular staining for interferon‐γ (IFNγ) prior to flow cytometric analysis. **(a)** Representative staining of IFNγ *versus* CD69 on NK cells (NK1.1^+^ CD3^–^). **(b)** Pooled data representing the percentage of NK cells positive for IFNγ or CD69. The graphs show the means ± s.e.m., *n* = 5 in two separate experiments. ***P < 0.001 *versus* medium alone controls. TLR2, Toll‐like receptor 2.

To determine whether the NK cells recruited in response to TLR2 activators could be activated, freshly isolated peritoneal or spleen cells from FSL‐1 or saline‐treated mice were cultured *ex vivo* with IL‐12 and IL‐18, a combination known to activate NK cells.^27^ Peritoneal NK cells from either saline or FSL‐1‐treated mice were efficiently activated by IL‐12 and IL‐18 to express IFNγ and CD69 (Figure 7).

## DISCUSSION

TLR2 activation is critical for initiating and shaping both early innate and sustained adaptive immune responses in multiple contexts including infectious diseases, cancer and TLR2‐mediated immunotherapy. Interestingly, in many of these settings early recruitment of NK cells occurs. This can result in NK cell direct cytolytic activity and/or cytokine production and also facilitates interactions with dendritic cells, T and B cells to regulate the developing adaptive immune response. Therefore, understanding the mechanisms of NK cell recruitment in situations where TLR2 activation occurs is important for understanding the mechanism underlying TLR2‐initiated immune responses and determining how to potentially modulate this process therapeutically. In this report we demonstrate that NK cells were recruited to the mouse peritoneum in response to TLR2 activation and that this recruitment was dependent on the classical TLR2–MyD88 pathway. The recruited NK cell numbers remained stable in the peritoneal cavity between 16 and 48 h after TLR2 activation, but NK cells did not acquire an activated phenotype even though peritoneal NK cells respond to become activated when exposed to IL‐12 and IL‐18 (Figures 6 and 7). In addition, there was a moderate, but significant decrease in the immature CD11b^–^CD27^+^ population and an increase in the more mature CD11b^+^CD27^lo^ NK cell subset. TLR2 activation induced multiple chemokines, with two CCR2 ligands, CCL2 and CCL7, showing the highest levels within the peritoneum. NK cell migration to the peritoneal cavity was absent in CCR2 knockout mice, but significant in CCR4‐, CCR5‐ and CXCR3‐deficient mice. The migration of adoptively transferred CCR2‐deficient NK cells to the peritoneum was also significantly lower than the migration of wild‐type NK cells, indicating a cell‐intrinsic component of CCR2‐mediated NK cell migration.

Chemokine involvement in NK cell recruitment has been analyzed in several models of infectious and inflammatory stimuli. Interestingly, although several chemokine receptors can be involved in NK cell recruitment (e.g. CXCR3, CXCR4, CXCR6, CCR2 and CCR5), the NK cell response to a specific cause of inflammation at a specific site is often more dependent on a single chemokine receptor. For example, recruitment of NK cells to the lymph node during cowpox virus infection is dependent on CXCR3,^23^ whereas NK cell recruitment in response to peritoneal vaccinia virus infection is dependent on CCR2.^34^ Recruitment of NK cells to the lung during *Aspergillus fumigatus* or influenza virus infection is also dependent on CCR2^22^, ^35^ and CCR5 is critical for the NK cell response following vaginal herpes simplex virus type 2 infection or in response to *T. gondii*.^24^, ^36^ The peritoneal chemokine response following TLR2 activation by FSL‐1 produced large amounts of ligands for CCR2 including CCL2 and CCL7 (close to 4500 and 7500 pg mL^–1^, respectively), with fourfold to sevenfold less of the CXCR3 ligand CXCL10, and 20‐ to 35‐fold less of individual CCR5 ligands such as CCL4 (Figure 3b). This dominance of CCR2 ligand production was reflected in the CCR2 dependence of peritoneal NK cell migration (Figures 4 and 5). Therefore, significant activation of TLR2 during peritoneal infections or tumor growth could result in similar NK cell recruitment. NK cell recruitment was not detected during the early neutrophil‐dominated FSL‐1 response (at 4 h, data not shown), but was evident at 16 h during the time that inflammatory monocytes are also recruited. The CCR2‐dependent NK cell recruitment that we describe ensures that this subset of NK cells will also be accompanied by the CCR2‐dependent influx of inflammatory monocytes (Figure 4 and ^37^), thus promoting a coordinated multipronged immune response. Although CCR2^+^ NK cells have been previously described and were protective in an *A. fumigatus* lung infection model,^22^ a more detailed functional characterization is still required. Interestingly, the peritoneal NK cell response in a cecal ligation and puncture model was CXCR3 dependent and resulted in activated peritoneal NK cells, expressing CD69 and producing IFNγ.^38^ In contrast to peritoneal vaccinia virus infection and several bacterial infection models such as *Haemophilus influenzae* and *Escherichia coli* where NK cells are protective,^34^, ^39^ NK cell recruitment and activation are detrimental in the cecal ligation and puncture model because NK cell depletion prior to cecal ligation and puncture markedly increased survival and decreased inflammation.^40^ Continued characterization of NK cell subsets could identify properties that are beneficial in certain immune responses, but harmful in a different context.

We observed that the initial wave of recruited NK cells after TLR2 activation does not appear to be activated. There are moderate changes in populations defined by CD11b and CD27 expression and changes in the percentage of NK cells expressing other receptors, including Ly49H, even in the absence of its known ligand, the m157 protein from murine cytomegalovirus (Figure 6). Other studies have reported that direct TLR2 engagement is important for NK cell activation, including during the response to virus infections (e.g. vaccinia and herpes simplex virus) or ligands such as the protein‐bound polysaccharide krestin.^14^, ^41^, ^42^ Virus infection and complex ligands could provide broader and more sustained signaling to facilitate TLR2 involvement in NK cell activation. Our results from the administration of a single dose of the specific TLR2 ligand FSL‐1 suggest that NK cell recruitment and activation are, at least initially, uncoupled. It is possible that FSL‐1 administration could prime NK cells to respond better to subsequent activation signals, a response we would not have detected *ex* *vivo* given the high concentrations of IL‐12 and IL‐18 (Figure 7). *In vivo,* NK cells recruited after TLR2 activation would be awaiting an ongoing immune response that generates type I IFNs in addition to other cytokines such as IL‐2, IL‐12, IL‐15 and IL‐18 that would activate NK cells (Figure 7 and Freeman *et al*.^27^). An environment rich in IL‐10 and transforming growth factor‐β would conversely favor the development of regulatory NK cells.^43^ Alternatively, extended TLR2 activation or other targeted therapeutics could serve to activate and differentiate the NK cells into more cytotoxic or regulatory phenotypes.^14^, ^44^

Because TLR2 ligands are known to influence the induction of a broad, effective immune response by activating several cell types, TLR2 modulation is currently being explored in the contexts of infectious diseases, vaccination and cancer therapy. Whereas TLR2 antagonists are primarily being utilized to prevent symptoms of sepsis,^12^ a wide variety of TLR2 agonists have been used to amplify the immune response in several models of infection, vaccination or antitumor immunity.^3^, ^12^, ^45^, ^46^ In many cases, TLR2 activation of dendritic cells improves their ability to activate T cells and correlates with an improved immune response,^47^ but direct T‐cell and NK cell activation can also be important.^41^ Clinical trials of TLR2 agonists as anticancer therapeutics have been limited but have suggested efficacy in patients with pancreatic and colorectal cancer.^45^, ^48^ The mechanisms of antitumor action following TLR2 agonist therapy, primarily based on preclinical data, have included NK cell activity as important in limiting tumor growth.^41^, ^49^ Although we have previously observed a mast cell dependence in TLR2‐mediated antitumor activity and NK cell recruitment,^13^ peritoneal NK cell recruitment following TLR2 activation was not dependent on mast cells (Supplementary figure 4). The recruitment of NK cells to the peritoneum that we observed following FSL‐1 treatment could be particularly relevant in colon and ovarian cancer, in which the peritoneum is often the site of secondary tumor development.^25^, ^26^ TLR2 agonist treatment to recruit NK cells, combined with additional components that activate NK cells (e.g. type I IFNs or IL‐12 and IL‐18), could potentiate NK cell‐mediated tumor clearance. Although we focused more intently on characterizing the peritoneal recruitment of NK cells in response to FSL‐1, we also observed that the triacyl lipopeptide Pam_3_CSK_4_ resulted in similar chemokine gene induction and similarly increased NK cell recruitment (Figures 1 and 3). Interestingly, differences in TLR2 agonists have been described to alter their ability to activate NK cells.^44^, ^50^ Therefore, a systematic comparison of TLR2 agonists could identify which TLR2 agonists optimize the aspects of NK cell recruitment and activation necessary for maximizing a specific immune response.

The observation that activation of the pattern recognition receptor TLR2 quickly recruits NK cells to an inflammatory site underscores the importance of both TLR2 and NK cells as critical components of innate immunity during bacterial, fungal and viral infections. This provides a potential mechanism to optimize the therapeutic functions of some antibody‐based cancer therapies, by locally mobilizing a key population of effector cells which participate in antibody‐dependent cellular cytotoxicity. Moreover, therapeutic success of TLR2 agonists to combat infections and tumors may be improved if the conditions required for optimal recruitment and activation of NK cells are taken into consideration.

## Methods

### Mice

C57BL/6, B6.Cg‐*Kit* ^W‐sh^/HNihrJaeBsmJ (*Kit^W‐sh/W‐sh^*), B6.129‐*Tlr2* ^tm1Kir^/J (*Tlr2^–/–^*), B6.129S1‐*Nod2^tm1Flv^*/J (*Nod2^–/–^*), B6.129(Cg)‐*Ccr2^tm2.1Ifc^*/J (*Ccr2^–/–^*) and B6.129S1‐*Ccr5^tm1Kuz^*/J (*Ccr5^–/–^*) were purchased from The Jackson Laboratory (Bar Harbor, ME) and bred in‐house. CXCR3‐deficient animals (*Cxcr3^–/–^*) were generously provided by Dr Brent Johnston (Dalhousie University, Halifax, Canada) and bred in‐house. CCR4‐deficient animals (*Ccr4^–/–^*) were generously provided by Dr Steven Kunkel (University of Michigan, Ann Arbor, Michigan) and bred in‐house. *Myd88* knockout animals were generously provided by Dr S. Akira (Osaka University, Osaka, Japan) and bred in‐house. *Nod2* and *Tlr2* knockouts were bred to produce double knockouts for both *Nod2* and *Tlr2*. Female mice aged 7–14 weeks were used in all experiments. Mice were housed under specific pathogen‐free conditions with food and water provided *ad libitum*. All experiments followed the guidelines provided by the Canadian Council on Animal Care and were performed according to protocols approved by the Animal Research Ethics Board of Dalhousie University (Halifax, Canada).

### Analysis of peritoneal inflammation

Mice were injected intraperitoneally with FSL‐1, Pam_3_CSK_4_ (EMC Microcollections, Tübingen, Germany) or *S. aureus* PGN (FLUKA, Oakville, Canada) at the indicated doses in 100 μL saline. At 16 or 48 h postinjection, the mice were killed and peritoneal contents harvested by lavage with 4.5 mL ice‐cold phosphate‐buffered saline (PBS), 5 mm ethylenediaminetetraacetic acid and 0.5% bovine serum albumin (all MilliporeSigma, Oakville, Canada). Lavage fluid was aliquoted and stored frozen for later analysis. The cells were counted and stained for flow cytometric analysis or used for RNA preparation. Spleens were also harvested from mice and processed for flow cytometric analysis.

For adoptive transfer experiments, splenocytes from wild‐type, *Ccr2^−/−^* or *Ccr5^−/−^* mice were depleted of B cells, neutrophils and macrophages by passage over nylon wool. This NK cell‐enriched population was labeled with CFSE (MilliporeSigma), resuspended in PBS and 10 million cells were injected intravenously into wild‐type recipients. These mice were immediately injected intraperitoneally with 100 μL saline or FSL‐1 (1 μg). After 16 h the peritoneal contents and spleens were harvested as described earlier, the total cells were counted and cells were analyzed by flow cytometry to determine the percentage of CFSE^+^ NK cells. Flow cytometry analysis of the injected cells was used to determine the number of NK cells injected (5.96–12.1 × 10^5^; see Supplementary figure 3). The numbers of CFSE^+^ NK cells in the peritoneum and spleen were normalized by calculating the number of CFSE^+^ NK cells detected per 10^5^ NK cells injected in each mouse [(number of total cells counted × percentage of CFSE^+^ NK cells among all cells as determined by flow cytometry)/the value of 10^5^ NK cells injected].

### Flow cytometric analysis

Specific leukocyte populations in the peritoneum and spleen were identified by staining with panels of the following reagents (clone names in parentheses): NK1.1 (PK136), CD3 (145‐2C11), CD4 (GK1.5), CD8 (53‐6.7), CD11b (M1/70), CD19 (6D5), CD90.2 (30‐H12), F4/80 (BM8), Gr‐1 (RB6‐685), Ly6G (1A8), Ly6C (HK1.4), Siglec‐F (E50‐2440) and streptavidin conjugates. Subsets identified included NK cells (NK1.1^+^CD3^–^), neutrophils (CD11b^+^F4/80^–^Ly6G^+^) and resting macrophages (CD11b^+^F4/80^hi^Ly6G^–^; see Supplementary figure 1). Phenotypic characterization of peritoneal NK cells [gated as in Supplementary figure 1, but also including a gate for single cells (>99%) and viability dye‐negative cells (>97%)] was assessed with antibodies specific for CD49b (DX5), CD11b (M1/70), CD27 (LG.7F9), NKG2D (CX5), KLRG1 (2F1), CD69 (H1.2F3), CD90.2 (30‐H12) and Sca‐1 (D7). All antibodies were diluted in PBS + 2% fetal bovine serum (FBS) (Thermo Fisher Scientific, Mississauga, ON, Canada) + 20 mm NaN_3_ (MilliporeSigma) and incubations were for 30 min on ice. Prior to staining, cells were incubated with PBS + 5% FBS + 20 mm NaN_3_ for 10 min to reduce nonspecific binding. Isotype controls and/or fluorescence minus one staining for all antibodies with the appropriate fluorochromes were utilized to determine background staining. Antibodies were obtained from BD Biosciences (Mississauga, Canada), BioLegend (San Diego, CA) and eBioscience (San Diego, CA). Stained samples were fixed with 1% paraformaldehyde (MilliporeSigma) and acquired with a BD FACSCalibur, FACSAria III or LSR Fortessa (BD Biosciences, San Diego, CA). Data analysis was performed with FCS Express version 3 software (De Novo Software, Los Angeles, CA) or FlowJo version 10 (FlowJo, Ashland, OR).

### *Ex vivo* NK cell activation

Peritoneal cells and spleens were isolated from C57BL/6 mice that had been injected intraperitoneally 16 h earlier with saline or FSL‐1 (1 µg). Following red blood cell lysis of the splenocyte preparation, splenocytes and peritoneal cells were washed with PBS + 2% FBS, resuspended at 1 × 10^6^ cells mL^−1^ in RPMI (Thermo Fisher Scientific) + 10% FBS and cultured for 1 h at 37°C. Nonadherent cells were removed and cultured further in either medium alone or medium with 10 ng mL^−1^ of both IL‐12 and IL‐18 (BioLegend).^27^ After 5 h, a Brefeldin A–Monensin cocktail was added to each well at the recommended concentration (BD Biosciences) and the cells were cultured for an additional 1 h. Cells were harvested and labeled with fixable viability dye eFluor 450 (Thermo Fisher Scientific) before surface staining for CD3 (145‐2C11), NK1.1 (PK136) and CD69 (H1.2F3) as described earlier. Cells were fixed in 1% paraformaldehyde for at least 30 min before incubating with BD Cytofix/Cytoperm for 20 min at 4°C. The cells were stained with anti‐IFNγ (XMG1.2), fixed in 1% paraformaldehyde for at least 30 min and transferred into PBS + 2% FBS + 20 mm NaN_3_ prior to acquisition and analysis as described earlier to identify NK cells and assess expression of CD69 and IFNγ.

### ELISA and Luminex assays

The concentrations of chemokines in the peritoneal lavage were determined by multiplex Luminex assays and ELISA. A commercially available Luminex assay panel (eBioscience) was used according to the manufacturer’s instructions to measure CXCL1, CXCL2, CXCL10, CCL2, CCL3, CCL4, CCL5, CCL7 and CCL11. ELISAs were also performed to measure CCL2 (PeproTech, Rocky Hill, NJ), CCL3 and CXCL10 (all R&D Systems, Minneapolis, MN). The reported values were adjusted for the dilution of the endogenous peritoneal cavity fluid (assumed to be 100 μL in all mice^28^) by the lavage fluid (4.5 mL).

### Quantitative polymerase chain reaction

Total RNA was extracted using the RNeasy Plus Mini Kit (Qiagen, Mississauga, Canada). Genomic DNA was depleted and complementary DNA was generated using the QuantiTect Reverse Transcription Kit (Qiagen). To assess the expression of multiple genes in the peritoneal cavity cells of saline *versus* FSL‐1‐treated C57BL/6 animals, PCR arrays (Qiagen) analyzing 80 chemokine and cytokine genes were utilized according to the manufacturer’s instructions. To assess the relative expression of several individual genes of interest, commercial primer pairs (Qiagen) for *Gapdh*, *Hprt1*, *Ccl2*, *Ccl3, Ccl4*, *Ccl7* and *Cxcl10* were used. The QuantiFast SYBR Green PCR Kit (Qiagen) was used to perform duplicate quantitative PCRs in a Bio‐Rad CFX 96 (Mississauga, Canada). Data were analyzed using Bio‐Rad CFX Manager Software version 3.1. Statistical analysis was performed on normalized data (2^ΔCq^), comparing saline *versus* activated expression of all genes with multiple *t*‐tests and Holm–Šídák correction. The fold induction of gene expression in saline *versus* treated animals was calculated using the ΔΔCq method.

### Statistical analysis

Comparisons of the cell numbers in the peritoneum were performed by one‐way analysis of variance (ANOVA), followed by Dunnett’s multiple comparison test of PGN, FSL‐1 and Pam_3_CSK_4_ treatments *versus* saline animals. Cell numbers in FSL‐1‐treated Ccr2^–/–^, Ccr4^–/–^, Ccr5^–/–^, Cxcr3^–/–^ and C57BL/6 mice *versus* their respective saline injected controls were compared by Student’s *t*‐tests. Comparisons of the number of CFSE‐labeled NK cells migrating to the peritoneum following adoptive transfer were performed by one‐way ANOVA and Holm–Šídák multiple comparison tests. Analysis of *ex vivo* NK cell activation and analysis of C57BL/6 *versus* *Kit^W‐sh/W‐sh^* mice were performed with two‐way ANOVA followed by the Holm–Šídák multiple comparison test. Peritoneal cytokine levels were analyzed by *t*‐tests of saline *versus* FSL‐1‐injected animals. *P*‐values less than 0.05 were considered significant. All statistical analyses were performed with two‐tailed tests using Prism version 6 (GraphPad Software Inc., La Jolla, CA).

## Conflict of Interest

The authors have no conflicts of interest to declare regarding the work reported in this manuscript.

## Supporting information

Supplementary figures 1–4
