# Supplementary material for: Toll‐like receptor 2 activation induces C–C chemokine receptor 2‐dependent natural killer cell recruitment to the peritoneum
Source: Immunol Cell Biol. 2020 Sep 9;98(10):854–67. doi: 10.1111/imcb.12379 (PMC7754274; doi:10.1111/imcb.12379)
Supplement: Supplementary file 1 — Supplementary figures 1–4 [file IMCB-98-854-s001.pdf]

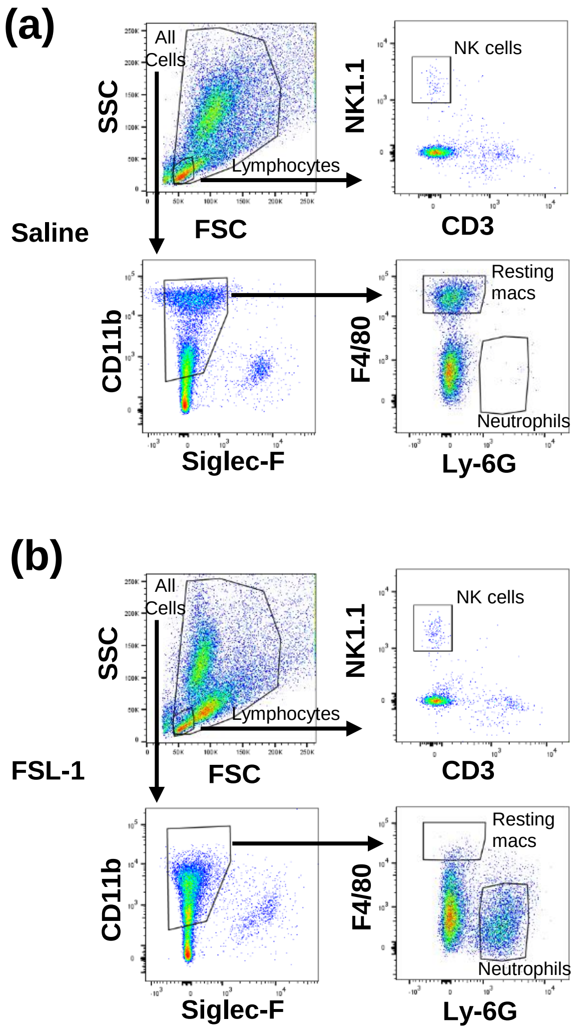

Supplementary figure 1. Representative gating to identify NK cell and neutrophil recruitment in response to TLR2 activation. Shown are representative flow cytometry profiles used to identify neutrophils and NK cells in the peritoneum of **(a)** saline injected or **(b)** FSL-1 (1.0 μg) injected C57BL/6 mice. After 16 hours the peritoneal contents were harvested by lavage and cells were labelled with specific antibodies to identify NK cells and neutrophils as shown. NK cells were identified as NK1.1^{+}CD3^{-} cells after first gating on only the lymphocyte population. To identify neutrophils, an FSC/SSC gate that included all cells was further gated on CD11b^{+}Siglec-F^{-} cells. The CD11b^{+} cells were analyzed for F4/80 and Ly-6G expression to identify Ly-6G^{+}F4/80^{-} neutrophils. The resting macrophage (macs) population (Ly-6G^{-}F4/80^{+}) is also shown as the major myeloid population in saline treated animals.
