# Supplementary material for: Toll‐like receptor 2 activation induces C–C chemokine receptor 2‐dependent natural killer cell recruitment to the peritoneum
Source: Immunol Cell Biol. 2020 Sep 9;98(10):854–67. doi: 10.1111/imcb.12379 (PMC7754274; doi:10.1111/imcb.12379)
Supplement: Supplementary file 2 [file IMCB-98-854-s002.pdf]

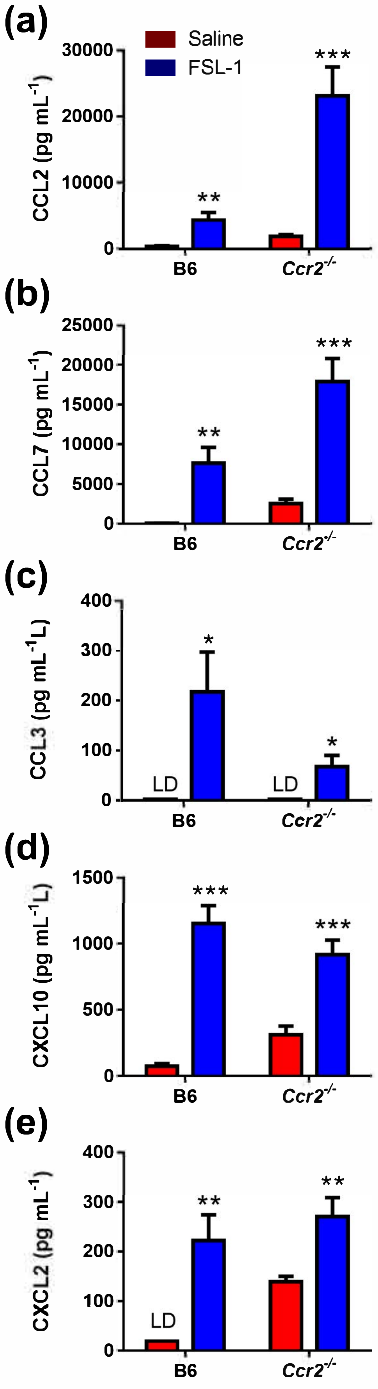

Supplementary figure 2. Chemokine levels in response to FSL-1 in *Ccr2*-deficient mice. Peritoneal lavages from wild-type mice (B6) or *Ccr2*-deficient mice that had been injected i.p. with saline or FSL-1 (1 μg) 16 hours before sacrificing, were analyzed for the presence of several chemokines as indicated. The results shown are the means ± SEM, n=5-11 in 2 or 3 separate experiments. *, $P < 0.05$; **, $P < 0.01$; ***, $P < 0.001$ vs. saline treated control in each strain.
