# Supplementary material for: Toll‐like receptor 2 activation induces C–C chemokine receptor 2‐dependent natural killer cell recruitment to the peritoneum
Source: Immunol Cell Biol. 2020 Sep 9;98(10):854–67. doi: 10.1111/imcb.12379 (PMC7754274; doi:10.1111/imcb.12379)
Supplement: Supplementary file 3 [file IMCB-98-854-s003.pdf]

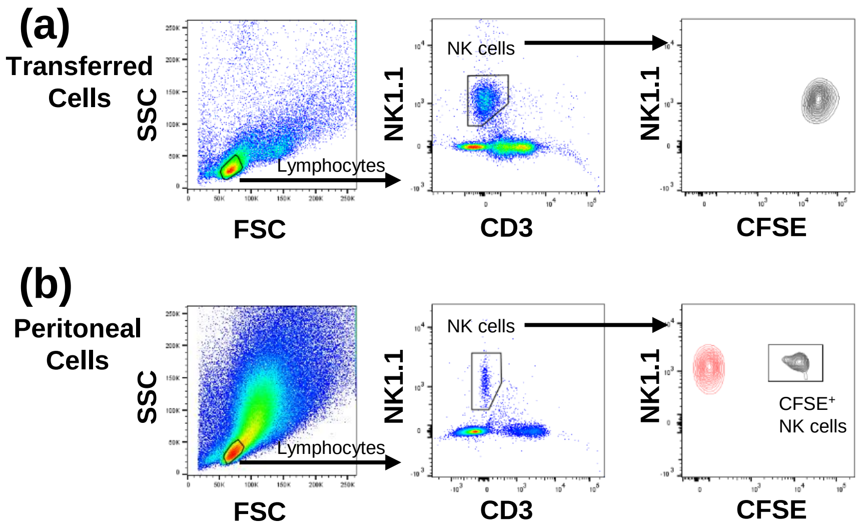

Supplementary figure 3. Representative gating to follow recruitment of adoptively transferred CFSE$^+$ NK cells in response to peritoneal TLR2 activation. **(a)** Spleen cells from C57BL/6, Ccr$^{2-/-}$, or Ccr$^{5-/-}$ mice were enriched for NK cells by reduction of B cells and macrophages. The cells were labeled with CFSE and stained for NK1.1 and CD3 to identify the NK1.1$^+$CD3$^-$ NK cell population prior to i.v. injection into recipient C57BL/6 mice. These mice were immediately injected i.p. with saline or FSL-1 (1 mg) and the peritoneal contents and spleen were harvested 16 hours later. **(b)** A representative flow cytometry staining for peritoneal cells from an FSL-1 injected mouse that received wild-type CFSE-labeled cells. NK cells were identified as NK1.1$^+$CD3$^-$ cells in the lymphocyte gate prior to identifying the percentage of CFSE$^+$ NK cells.
