# Supplementary material for: Toll‐like receptor 2 activation induces C–C chemokine receptor 2‐dependent natural killer cell recruitment to the peritoneum
Source: Immunol Cell Biol. 2020 Sep 9;98(10):854–67. doi: 10.1111/imcb.12379 (PMC7754274; doi:10.1111/imcb.12379)
Supplement: Supplementary file 4 [file IMCB-98-854-s004.pdf]

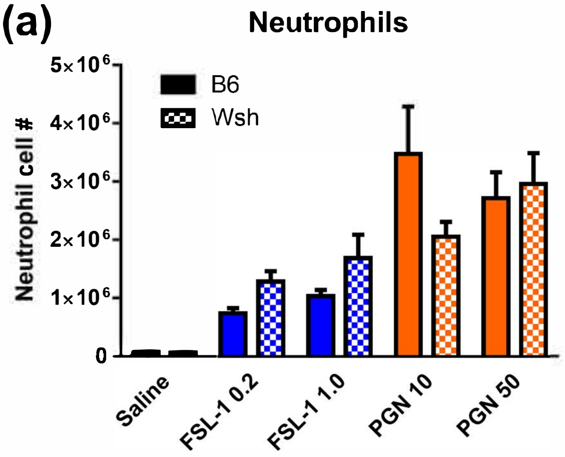

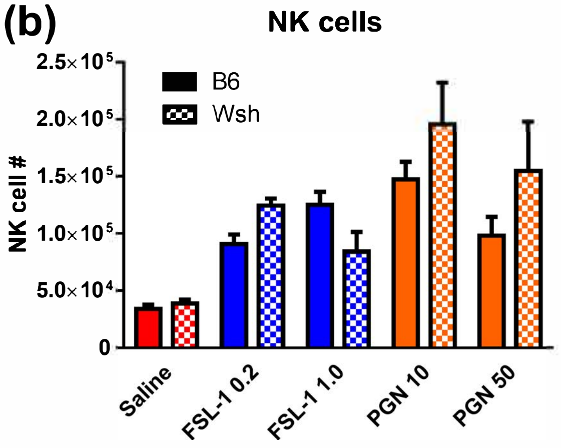

Supplementary figure 4: NK cell recruitment in mast cell-deficient mice. C57BL/6 (B6) or $Kit^{W\text{-}sh/W\text{-}sh}$ (Wsh) mice were injected i.p. with saline, FSL-1 (0.2 or 1.0 μg), or PGN (10 or 50 μg). After 16 hours the peritoneal contents were harvested by lavage, the total cells were counted, and the percentages of **(a)** neutrophils and **(b)** NK cells were identified by flow cytometric analysis. The graphs shown are the means ± SEM, n=4-32 in 2 to 7 experiments for all activation conditions. No significant differences were found between each pair of values for B6 vs. Wsh as analyzed by 2-way ANOVA with a Holm-Sidak multiple comparison test.
